# Homicides and Suicides by Mentally Ill People

**DOI:** 10.1100/tsw.2003.51

**Published:** 2003-08-02

**Authors:** Said Shahtahmasebi

**Affiliations:** School of Mathematics and Statistics, Faculty of Health and Sciences, Christchurch Polytechnic Institute of Technology, POBox 540, Christchurch, New Zealand

**Keywords:** community records, health informatics, life events, United Kingdom

## Abstract

In 1992, following consultations with the Royal College of Psychiatrists, the confidential inquiry into homicides and suicides by mentally ill people was set up by the United Kingdom Department of Health. The inquiry collects detailed information on contact with secondary mental health services by means of a questionnaire from clinical audit or information departments from these organisations. In Leeds, however, a wider range of available records including Coroner Reports, police, social, educational, and all health records were consulted. This resulted in a series of health/life event histories of suicide cases that had been in contact with psychiatric services. This paper presents an exploratory analysis of these data. The Leeds suicide cases formed less than one-third of all suicide cases in Leeds; the remainder had not come into contact with psychiatric services. This proportion is consistent with the U.K. national figures. Records show that 46% of the sample's first contact with the psychiatric services was through a first failed attempted suicide. Other results include the role of prescribed drugs in repeat suicide attempts, education levels, and employment stability. It is concluded that the link between mental illness and suicide is questionable. Life event history type data on all suicide cases is desperately required to study suicide as a social process.

## INTRODUCTION

Suicide has received a great deal of attention from various scientific disciplines. At the time of writing this report, a restricted Medline search on the keyword "suicide" since 1990 retrieved 18,411 (total entries at PubMed was 33,633), a similar search on CINAHL yielded 3,979 published articles, and a search on ProQuest yielded 100,000 items. Suicide is socially unacceptable in most modern societies and the literature suggested many different attempts to explain suicide and its causes; for example, suicide among patients with affective disorders[1], suicide and psychiatry and physical morbidity[2], suicide amongst university students[3], suicide and women[4], suicide and youth[5,6], suicide in old age[7], religion and suicide[8], cholesterol levels and suicide[9,10], most citing mental disorder, social, educational, and life events as risk factors[11]. For example, Draper[7] cites depression, pain, grief, loneliness, alcoholism, and career stress as some of the treatable risk factors in old age suicide. Most of the literature was based on the studies of self-harm and failed suicide attempts who by law receive a psychiatric assessment and will have a psychiatric record. However, this group forms only the smaller proportion of the suicide population, whilst studies of attempters add complexities by introducing bias[12]. There are multiple factors that contribute to the suicide rate[13,14,15].

Within the suicide literature, despite the large volume of work, criticisms have been aired that our knowledge of suicide is based on scant scientific evidence[12,16,17]. Study design and quality of data have been cited as being responsible for the ambiguities in the evidence. For example, one study[18] suggested a population age-specific rate of 3.5% for sufferers of depression as opposed to 15% in other studies. Some have suggested that medical intervention (antidepressant prescription) may not be the solution in reducing suicide rates, while Hall et al.[19] — using data from antidepressant sales and prescriptions — concluded that antidepressant prescription was a main contributing factor in reducing the suicide rate. However, the common consensus is that suicide is a major cause of (potentially) preventable death.

The major problem with suicide research is that the key informants are no longer available to provide insight into the events. On the other hand, prospective designs require large samples over long periods of time, which are often ethically complex and financially prohibitive. For this reason, studies of suicide have mainly been retrospective using psychiatric records and surveys of significant others (next of kin), or the study of a subpopulation, e.g., those with failed attempted suicide or those with a history of self-harm. Such studies are limiting, as they are subjective and exclude the dynamics of life processes and may lead to spurious relationships and give undue emphasis to some other variables.

The main issue is, therefore, the availability of objective suicide data. Most countries have a system of record keeping for monitoring outcomes and resource allocation for community services. The quality of these records varies from service to service and country to country, but they are the only records collected during the lifetime of individuals.

In 1992, following consultations with the Royal College of Psychiatrists, the confidential inquiry into homicides and suicides by mentally ill people was set up in the U.K. by the Department of Health. The inquiry collects detailed information on contact with secondary mental health services by means of a questionnaire from clinical audit or information departments from these organisations[20]. In Leeds, however, a wider range of available records including coroner reports, all health, social, justice, and educational records were consulted. This resulted in a series of health/life event histories of suicide cases that had been in contact with psychiatric services. In most cases a health/life event history can be constructed.

Analysis of such data requires a well thought out methodology that incorporates the complexities of multiple time series data, which are both quantitative and qualitative. This paper reports results from the first round of exploratory analysis of these data providing a wider perspective for investigation and analysis of suicide.

## METHODS

A comprehensively designed proforma was used for the purpose of data extraction from the public and medical records. The data gathered included both quantitative and qualitative data. Demographic and socioeconomic information were generally of a quantitative nature whilst narration of ill-health, social, and educational histories were qualitative in nature. These are the subject of a further textual and data mining exercise and will be reported on completion.

However, to obtain an initial view of the distribution in the sample of childhood, adolescence, and education, the analysis was restricted to the presence or absence of a life/health event and its type. For example, a turbulent childhood, violence within the family, problems at school, and problems with educational development are used as proxy to indicate existence of problems during the growing up years. That is to say, the post coding was not based on any prior theoretical background or assumptions, and no attempt was made to investigate complex inter-relationships between various demographic and socioeconomic backgrounds and suicide. The main reason for these proxy variables is that all the cases were suicide cases, they all had at least one contact with psychiatric services, and the sample excludes those cases without contact with the services.

In this paper, we report results from the preliminary analysis of the quantitative data. A limitation of this study was that we have no knowledge about the cases of suicide that had no contact with psychiatric services.

## RESULTS

### Demographic Background of the Sample

During the period 1993–96, 48 cases of suicide that had received psychiatric care had been identified. These are shown in Table 1 by type of coroners' verdict and gender. The age/sex distribution in the sample was similar to other studies reporting suicides in the North of England[21,22]. There were more men in the sample than women; 30 men (mean age 39, 95% CI 35–43) and 18 women (mean age 49, 95% CI 43–56). The age difference between the sexes appeared to be statistically significant (t = 2.84, $p < 0.01$).

**TABLE1**
**Identified Self-Harm Fatality while Under Psychiatric Care**

| | Male | Female | Total |
|---|---|---|---|
| Suicide | 13 | 5 | 18 |
| Open | 16 | 10 | 26 |
| Misadventure | 1 | 2 | 3 |
| Not Clear | | 1 | 1 |
| Total | 30 | 18 | 48 |

### Social Background of the Sample

Initial assessment of the data suggested that the majority of the cases (65%) may have had a reasonable childhood, as there were no records of any major events during those years. Around 17% had a record of unstable childhood history (e.g., trauma, bullying at school, divorcing parents), 8% had records of other major childhood problems (e.g., exposure to violence), and the childhood/adolescence of the remaining 10% of the sample was unknown.

Of those with no recorded events during childhood, 10% developed an unstable adolescence and only one case in this group had a more turbulent adolescence with a police/prison record. Of those with an unstable childhood, 50% continued to have an unstable adolescence, 25% developed problems with the police or had a prison record, one case was referred to the child psychiatric unit, and only one case appeared with no record of any major life event during adolescence.

The remaining 8% included two cases of childhood exposure to violence within the family, of which one had police records and a prison record and the other appeared without any major life events during adolescence; one case received special schooling in childhood and developed an unstable adolescence; and, finally, one case with a childhood history under the care of social services had an apparently uneventful adolescence.

## Education and Employment History

The majority of the sample had a poor educational history; around 71% of the sample appeared to have left school without any qualifications. This is quite a large proportion of the sample and is likely to be unrepresentative of the general population of Leeds. For example, a survey of Leeds employees[23] found that 27% of their workforce had no qualification. The overall unemployment rate in Leeds for the years 1994 and 1995 was reported to be 8.5 and 7.6%, respectively[24], but around 21% of the sample was recorded as long-term unemployed, whilst 19% had been through a variety of jobs (not being able to sustain a job for long), and only around 25% had managed to sustain at least one job for a period of time.

## First Contact with Psychiatric Services

Table 2 reveals that whilst 33% were self-referred to the psychiatric services (including a small proportion who had been referred by their GP), around 46% of the sample's first contact with psychiatric services was due to their first failed attempted suicide. In total, our sample generates a raw suicide mortality ratio of 2 per 100,000 population for 1993 and 1994, and 1.3 and 1.6 per 100,000 population for 1995 and 1996, respectively. Regional mortality data suggest a much higher mortality due to suicide with or without contact with mental health services. For example, the 1993 standardised mortality ratio (SMR) for suicide and undetermined injury for the Leeds Health Authority geographical area in the U.K. was around 16 per 100,000 population[25].

**TABLE 2**
**Method of Coming into Contact with Psychiatric Services**

| | Frequency in the Sample | Percentage (%) |
|---|---|---|
| Attempted Suicide | 22 | 46 |
| Addiction (Alcohol) | 6 | 13 |
| Other (e.g., self referral) | 16 | 33 |
| Not known | 4 | 8 |
| **Total** | **48** | **100** |

## Morbidity Background and Diagnosis

Our data suggested that psychosis was rare. However, the records indicated that around 65% had a medical history such as chronic physical illness; 42% of the sample had a medical history within the family. Similarly, 40% had a family history of psychiatric illness, e.g., one of the parents. From these records, 69% had a recorded diagnosis, 13% of which had a second, and 4%

had a third. Table 3 shows the distribution of diagnosis for the sample; 15 (31%) of the sample had no recorded diagnosis and 17% had a recorded depressive illness. The “other” category did not have a recorded diagnosis, but the word depression or depressive illness had been mentioned in the textual comments. It can be seen that it would be easy, if care is not exercised, to make a link between suicide and depression. Caution must be applied when interpreting such information because the sample is limited to only those with a psychiatric record; we do not have enough information about those without a record; we know that in the general population there are people with similar characteristics who do not commit suicide.

**TABLE 3**
**The Sample Distribution of Psychiatric Diagnosis**

| First Diagnosis | Frequency | Percentage |
|---|---|---|
| Depressive illness | 8 | 16.7 |
| Schizophrenia | 6 | 12.5 |
| Personality disorder | 3 | 6.3 |
| Alcoholism | 4 | 8.3 |
| Paranoid illness | 2 | 4.2 |
| Anxiety state | 1 | 2.1 |
| Other | 8 | 16.7 |
| No diagnosis | 1 | 2.1 |
| Nonrecorded | 15 | 31.3 |
| **Total** | **48** | **100** |

Furthermore, it should be noted that for about half the sample, a psychiatric assessment and diagnosis had been carried out following the suicide attempt. In most studies of suicide, self-harm and attempted suicide is assumed a precursor to a psychiatric diagnosis of a depressive illness. The implication may well be a feedback effect in which attempted suicide cases may have a higher rate of being diagnosed with a depressive illness. Some studies suggest that those who attempt suicide with intent to die are more likely to be depressed and feel hopeless[26]. Almost always we have no knowledge of the level of depression prior to first fatal or nonfatal suicide and are unable to distinguish past behaviour effect from the possible cumulative effects of failure to complete the process of suicide.

## Self-Harm History

Out of the 73% with a history of attempted suicide, 37% had attempted once before, 26% had attempted twice, and 23%, nine or more times before. Of the sample, 42% died within 6 months of previous unsuccessful attempts. This result was generally in agreement with the literature and comparable with the results from other studies (for example, see McKenzie and Wurr[27]). Since we know very little about the larger proportion of the cases of completed suicide first time, caution must be exercised in interpreting and attaching too much weight to this variable as a precursor of suicide.

## Prescribed Drugs

A further point of concern to the care service is that prescribed drugs were involved in combination or on their own in 61% of the cases that died as a result of overdose. This is rather disconcerting, as an analysis of method of previous attempts suggested that prescribed drugs were also involved in 57% of the cases. There have been a number of practice changes and legislation regarding drugs and prescriptions, e.g., the manufacturing of nontoxic drugs, limiting public access to a large number of household and commonly available painkillers. Despite such changes, our data suggested that there was a tendency to switch methods in subsequent suicide attempts until successful. An increasing suicide trend together with evidence of switching between methods suggested that whilst mortality due to drug overdose may have been contained, there had been no impact on the overall suicide attempt and suicide mortality rate. Similarly, recent data from New Zealand suggest that methods such as hanging (40%) and poisoning by other gases (28%) are becoming more common[28]. Prescription and drug management is clearly an important issue and needs to be considered strategically in conjunction with other policies.

## Alcohol Abuse

Around 15% of our sample had a history of alcohol abuse and had been referred to the addiction unit either through their GPs or through Psychiatric Services. Only in one case alcohol was determined as the main method of suicide.

## DISCUSSION

The preliminary study of case histories reveal a number of interesting points:

- The results raise the issue that we know very little about the majority of the suicide cases who succeed the first time and do not come into contact with psychiatric services[12,17].
- For a large proportion of the sample, a psychiatric diagnosis was made following the failed first attempted suicide (coming into contact with psychiatric service: 46% due to a failed suicide, 13% due to addiction, 33% due to self/GP referrals or other).
- Most of our sample appeared to have had at least one period of instability in their lives, had not finished school, had no qualifications, and had no stable occupation history.
- Medication appears to be a major issue in terms of compliance, actual benefits and clinical effect, and as a means of committing suicide.

It is important to note that although all the cases in our sample had come into contact with psychiatric services, some completed suicide whilst in care and some completed suicide within days (less than 5 days) of discharge following weeks of treatment. Others completed suicide within months, and only in one case, suicide was completed over 10 years since the previous attempt. The question for the service is whether medical treatment is merely a delaying mechanism or a potential remedy[29]. Other issues to be considered for service development are what factors govern the life processes that reduce resilience to suicide, health, and social care of those who do not come into contact with community services, and the quality of life following treatments and in between attempts. To address these issues, access to quality objective information is essential.

The aim of the original audit was to gather data on suicide cases under psychiatric care. As expected, therefore, most of the sample (67%) had a psychiatric diagnosis but only 17% diagnosed with a depressive illness. In line with other studies of this type[13], it appears that the outcomes of other variables — in particular life events and life processes — may, at least in part, explain suicide. Presently, the main issues may be considered to be twofold: (1) we do not know anything about suicide cases who were successful the first time and (2) critical evaluation of the evidence that link the various variables to suicide. For example, the population may have individuals with similar characteristics and circumstances — one becomes a suicide case whilst others do not. Indeed, the discrepancy in suicide literature may well be due to a lack of consideration to (2).

Few studies have investigated suicide using community medical records (GPs) in addition to psychiatric records[22]. Other similar studies based on community held records such as Torre et al.[13,30] reported suicide trends based on data gathered from hospital/casualty departments and the police force spanning over 6 years. The authors reported a number of demographic and social variables as well as mental illness being significantly related to suicide. They also reported some data limitation related to registration and coding of such official data. The Australian study[30] was concerned with youth suicide and risk taking deaths. Both studies suggest similar results. In addition to the individual's characteristics and life events, the studies are in agreement that a large proportion of cases have had no contact with the mental health services. Without access to the individuals who can provide insight into their process of decision making, this type of study involving data mining of all community records has the advantage of being objective.

The problems with surveys, follow up of attempters, and psychological "autopsies" are that they tend to be subjective and based on conjecture. Subjectivity is often due to several sources: the investigator, next of kin, and/or significant other. Studies of this kind attempt to build a psychological profile of the suicide case based on feelings, memories, perceptions, and recollections of other persons about the case. Subjectivity is responsible for spurious relationship in data[31]. The major flaws are the assumption that the suicide and suicide attempter populations are the same or similar, mental illness is presumed from the outset, and the study designs often do not match the aims and objectives of the study related to suicidal behaviour. On the other hand, data collected after the event (suicide) from significant others will be subject to spurious relationship through those providing the information. Furthermore, to study behaviour, individuals must be observed over time. For example, Beautrais[32], reported a case-control design, amongst other aims, to study the extent to which a series of risk factors including psychiatric illness contribute to the risk of suicidal behaviour. The main problem with this study is its methodology that gives rise to complex compounding and confounding in what is essentially a cross-sectional data set. Beautrais further reports that data including the psychiatric morbidity assessments were collected from significant others and medical records[32,33,34]. Like other western countries, suicide is a big issue in New Zealand and experience suggests a large proportion of cases not accessing the mental health services[35]. Study designs such as those of Beautrais[32], where subjectivity is allowed but not accounted for and where assessment of cases is based on interviewing of significant others after the event, artificially inflate the role of mental health[12]. It is not surprising that Beautrais[32,33] reports a high level of mental disorder, including depression, in the sample. However, the literature suggests that depression is much more common than has been reported[18,36]. The presence of depression would be expected in any sample taken from the population. Therefore, not much reliance can be placed on the results from such studies. Similarly, it is not surprising that the literature reports a link between low cholesterol levels and suicide[9,10]. Low cholesterol levels have been associated with depression that is regarded a suicide risk factor. What is needed is an investigation of the dynamics of suicide and whether the differential in the proportion of sufferers of depression is statistically significant between the various groups and that of the population.

The ambiguous relationship between mental illness and suicide[14,15] has naturally led the literature to question mental health service development and provision. For example, some authors question availability/accessibility of health care services[37], whilst others question the medical intervention such as antidepressant prescriptions[29]. Khan[29] analysed the FDA data on suicide rates for more than 71,604 patients who were treated in clinical trials and concluded that despite the exclusion of the patients exhibiting suicidal behaviour, suicide rates were high in these trials. On the other hand, Hall and colleagues[19] used data on antidepressant sales, prescriptions (from a sample of GPs), and mortality from suicide over the period 1991–2000 to conclude increasing antidepressant sales and prescriptions has led to falling suicide mortality. Such trend analyses adds more confusion than insight due to the time series being based on aggregate rather than individual data, difference in prescription between end-points only, and a lack of consideration to the seasonal and cyclic effects in suicide mortality rates over time[38]. Therefore, any positive or negative correlation between the trends in suicide rates and antidepressant prescription over a period of time may well be coincidental.

The literature's estimates of the suicide risk factors, i.e., the large number of individual characteristics and personal and social circumstances as suicide risk factors, suggests that we all run the risk of suicide at any point in our lifetime. Perhaps suicide research needs to widen its focus beyond the multidisciplinary approach of studying individuals and concentrate on a more holistic approach. In attempting to answer the question about what makes the population of suicide cases different, studies may focus on unmeasured latent individual and population variables, e.g., individual resiliency and its relationship to the dynamics of the society (economy, social support, moral and social values, environment). However, presently, a natural step to take would be to take a self-critical role and question our knowledge and understanding of suicide, accept suicide as the outcome of a decision-making process — processes are by nature dynamic[39].

Information is the most important currency in health care development and provision. More attention must be paid to the development and support of studies that lead to an improved health care information system, e.g., the design and utilisation of a unified database. One of the ways that a unified database may assist would be an expected improved communication between health care professionals. Improvements in communication could perhaps be achieved through an integrated electronic patients' record. Such a unified information system could facilitate the development and utilisation of available information on patients' case histories and enable risks to be "flagged".

## ACKNOWLEDGEMENTS

The author is grateful to the Leeds Community Mental Health Services NHS Trust for their permission to use the data that became available during the authors employment with the Trust; to Dr. John Wattis (MBCHB; DPM; FRCPsych), Medical Director, for his helpful comments on the original draft of the manuscript; and to Mr. Paul Newton, the Clinical Audit Manager for his assistance in accessing the data.

## BIOSKETCH

**Said Shahtahmasebi** is currently research methodology consultant and statistician at Christchurch Polytechnic Institute of Technology, Christchurch, New Zealand. His work experiences are mainly in the U.K. and cover a number of fields including public health, mental health, nursing, operational research, food and nutrition, and gerontology. His area of interests and expertise are related to longitudinal modelling of health related behaviour. He has set up the Good Life Project in New Zealand, which attempts to investigate food holistically.
